# Sestrin 2 suppresses cells proliferation through AMPK/mTORC1 pathway activation in colorectal cancer

**DOI:** 10.18632/oncotarget.17595

**Published:** 2017-05-03

**Authors:** Jin-Lai Wei, Min Fang, Zhong-Xue Fu, Shou–Ru Zhang, Jin-Bao Guo, Rong Wang, Zhen-Bing Lv, Yong-Fu Xiong

**Affiliations:** ^1^ Department of Gastrointestinal Surgery, The First Affiliated Hospital of Chongqing Medical University, Chongqing 400016, China; ^2^ Department of Emergency and Intensive Care Unit, The First Affiliated Hospital of Chongqing Medical University, Chongqing 400016, China; ^3^ Department of Thoracic Surgery, The First Affiliated Hospital of Chongqing Medical University, Chongqing 400016, China

**Keywords:** colorectal cancer, sestrin 2, AMPK, mTORC1, proliferation

## Abstract

Sestrin 2 is a conserved antioxidant protein that reduces reactive oxygen species (ROS) and inhibits mammalian target of rapamycin complex 1 (mTORC1). We previously showed that sestrin 2 is abnormally decreased in colorectal cancer (CRC). To elucidate the molecular mechanism behind the potential contribution of sestrin 2 to CRC, we used a lentiviral expression vector system to determine the effects of sestrin 2 overexpression on human CRC cells. We found that sestrin 2 overexpression decreased ROS production, inhibited cell growth, and stimulated apoptosis in two CRC cell lines. In parallel, expression of the proliferation marker PCNA was decreased, proapoptotic caspase 3, 7, and 9 levels were increased, and expression of the anti-apoptotic protein survivin was reduced. Sestrin 2 overexpression also activated the adenosine monophosphate-activated protein kinase (AMPK) pathway, and suppressed mTORC1 signaling. Treating CRC cells with compound C, an AMPK inhibitor, reversed or attenuated changes in proliferation, apoptosis, and signaling proteins of the AMPK/mTORC1 axis. In a xenograft mouse model, CRC growth was attenuated by sestrin 2 overexpression. These results suggest that sestrin 2 suppresses CRC cell growth through activation of the AMPK/mTORC1 pathway and induction of apoptosis, and could be a novel pharmacological target for the treatment of CRC.

## INTRODUCTION

Colorectal cancer (CRC) represents the third most commonly diagnosed cancer in men and the second in women worldwide, with over 1.4 million new cases and 694,000 deaths in 2012 [1]. The processes involved in the pathogenesis of CRC are complex, but current research suggests metabolic dysregulations are intricately linked to CRC progression [2]. Several biomarkers of metabolic pathways are related to CRC risk [3], and CRC could be considered as a metabolic disease [4]. Growing tumors rewire cellular metabolism of macromolecules to satisfy and even exceed the bioenergetic and biosynthetic demands of continuous cell growth [5], in a process facilitated by intrinsic genetic mutations and an abnormal tumor microenvironment [6]. Reactive oxygen species (ROS) comprise a diverse class of radical oxygen intermediates produced in all cells as a normal byproduct of metabolic processes. High levels of ROS produced during rapid proliferation of cancer cells can damage macromolecules, aggravating the progress of tumorigenesis [7]. Cancer cells counteract the detrimental effects of ROS production on metabolic processes by producing antioxidant molecules such as the proteins in the sestrin family.

Sestrins are encoded by an evolutionarily conserved, stress-inducible gene family and are involved in the regulation of cell survival in response to various stressful conditions such as oxidative or genotoxic stress [8–10]. Three sestrins, sestrin 1, sestrin 2, and sestrin 3, are expressed in mammals. Sestrin1 and sestrin 2 were classified as members of the growth arrest and DNA damage inducible gene family that regulates cell growth and viability under different cellular stressors [11–13], and are target genes of the tumor suppressor p53 [8, 14]. p53 is activated upon genotoxic and oxidative stress and inhibits cell proliferation and growth through induction of specific target genes, including sestrin 2 [15]. We have reported that the expression of sestrin 2 is down-regulated in both human CRC tissues and human CRC cell lines. Additionally, decreased sestrin 2 was associated with unfavorable prognosis and was an independent prognostic factor for CRC [16]. Although our previous results shed light on a novel function of sestrin 2 as a potential tumor-suppressor gene in CRC, the exact role of sestrin 2 in CRC remains unclear.

Recent research on the crystal structure of sestrin 2 proved that while the N-terminal domain participates in ROS reduction, the C-terminal domain contributes to the inhibition the mammalian target of rapamycin complex 1 (mTORC1) [17–18]. The mTORC1 branch of the mTOR pathway is a major driver of cell growth in mammals and is deregulated in many common tumors [19–20]. mTORC1 activation can promote cell survival through the activation of antiapoptotic proteins, contributing to tumor progression [21–22]. A study on prostate cancer has shown that activation of the mTORC1 substrates p70 ribosomal S6 kinase (p70S6K) increases protein levels of survivin, a member of the inhibitor of apoptosis protein (IAP) family [23]. In addition, sestrin 2 negatively regulates mTORC1 activity by inducing AMP-activated protein kinase (AMPK) activation under genotoxic stress [24]. Also, sestrin 2 interacts directly with AMPK in response to metabolic stress, thus helping to protect cells against stress-induced apoptosis [25]. AMPK is a heterotrimeric complex comprising two α (α1, α2), two β (β1, β2), and three γ (γ1, γ2, and γ3) subunits that serves as an essential regulator of metabolic activities controlling cell growth and proliferation [26–28]. An increased AMP:ATP ratio during energy stress leads to AMP-dependent phosphorylation of the catalytic α subunits [29]. This activates AMPK which then phosphorylates numerous substrates to restore energy homeostasis [26, 29]. AMPK functions as a tumor suppressor by inhibiting cell growth through suppression of mTORC1 signaling [30]. Accordingly, AMPK/mTORC1 signaling has been reported to be involved in carcinogenesis in various types of tumors including CRC [31].

Previous studies have shown that sestrin 1 and 2 proteins play a critical role in the inhibition of protein synthesis in response to ionizing radiation in breast epithelial MCF10A cells, through activation of AMPK and inhibition of mTOR [32]. Additionally, sestrin 2 induction in breast carcinoma MCF7 cells significantly inhibits the expression of growth regulatory proteins through a mechanism that involves stimulation of AMPK and mTOR inhibition [33]. All these data indicate that sestrin 2 expression or activity changes affect redox and metabolic homeostasis via AMPK/mTORC1 signaling, and could therefore contribute to tumorigenesis. We hypothesize that alterations in sestrin 2 expression contribute to CRC pathogenesis by modulation of the AMPK/mTORC1 pathway. In the present study, we investigated the effects of sestrin 2 on proliferation and apoptosis of human CRC cells, both *in vitro* and in a mouse xenograft model *in vivo*. Furthermore, we assessed the relationship between sestrin 2 and AMPK/mTORC1 signaling molecules using compound C, a selective AMPK inhibitor [34].

## RESULTS

### Analysis of sestrin 2 overexpression in CRC cells

Three experimental groups were established using cultured SW620 and LoVo CRC cells: 1) LV-sestrin 2 (cells with stable sestrin 2 overexpression); 2) LV-NC (negative control cells transduced with a non-targeted lentiviral vector); and 3) BC (untransfected, blank control cells).

**Figure 1:**
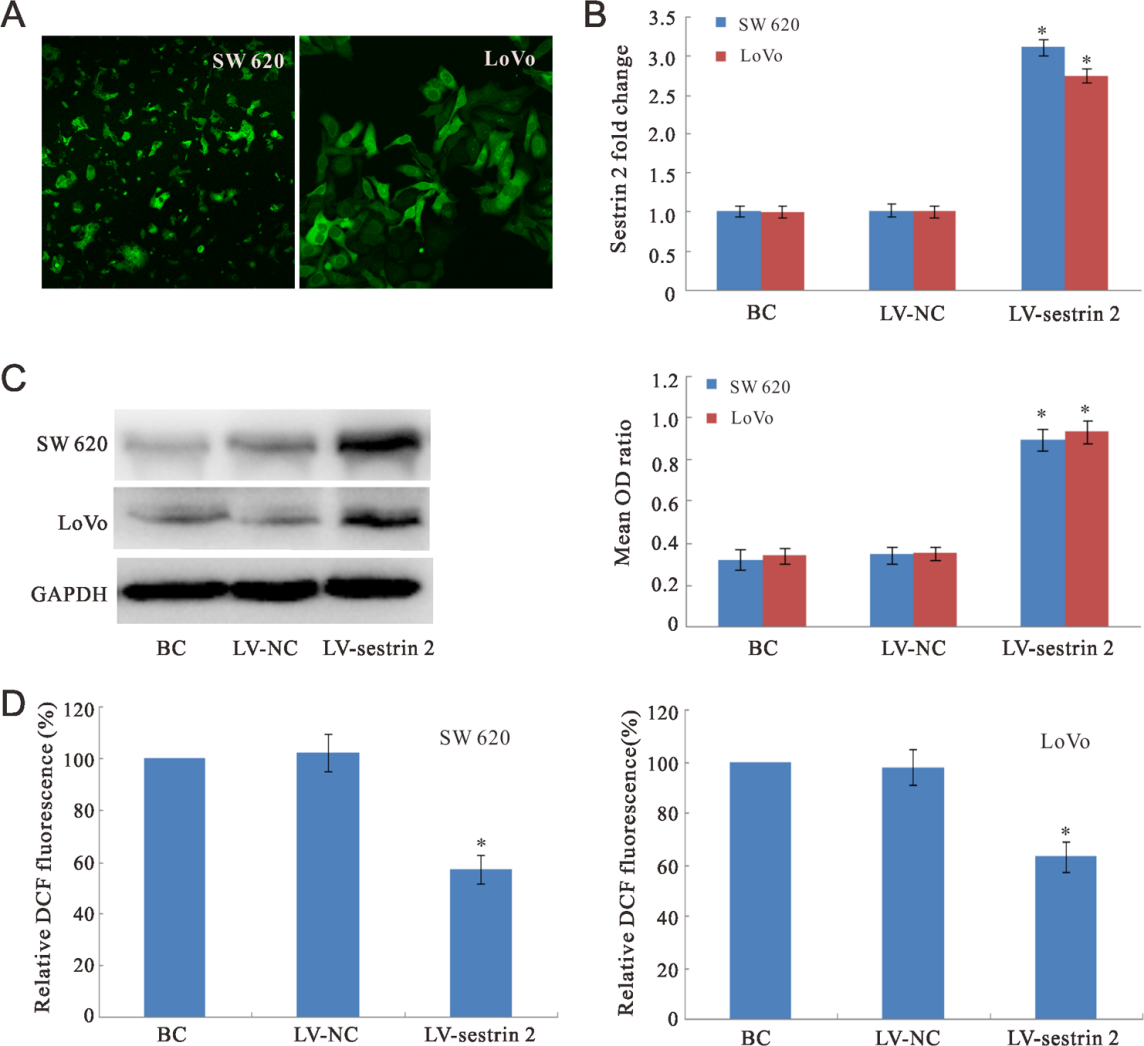
Sestrin 2 expression analysis and ROS assay in CRC cells (**A**) Immunofluorescence images showing EGFP expression in SW620 and LoVo cells after transduction of lentiviral vectors encoding sestrin 2. (**B**) qRT-PCR analysis of sestrin 2 mRNA fold changes in CRC cells. (**C**) Western blot analysis of sestrin 2 protein expression in CRC cells. (**D**) DCFH-DA probe staining. Intracellular ROS levels were significantly decreased in the LV-sestrin 2 group, compared with control BC and LV-NC groups. “BC”, blank control group; “LV-NC”, negative control group; “LV- sestrin 2”, sestrin 2 overexpression group. *p < 0.05 indicates statistically significant differences between LV-sestrin 2 group and BC or LV-NC control groups.

Lentivirus bearing EGFP was detected in SW620 and LoVo cells 3 days after transduction (Figure 1A). Analysis of sestrin 2 mRNA fold changes for the different experimental groups was performed using qRT-PCR 4 days after lentiviral transduction (Figure 1B). The expression of sestrin 2 mRNA was similar for both BC and LV-NC groups. Compared with these two groups, significantly higher (*p* < 0.05) sestrin 2 mRNA expression was detected in the LV-sestrin 2 group. Western blot analysis of SW620 and LoVo cells was performed using a sestrin 2-selective antibody 4 days after transduction to confirm that sestrin 2 protein levels were effectively increased (Figure 1C). Sestrin 2 expression was normalized by calculating the optical density (OD) ratios of the sestrin 2 bands to the corresponding β-actin bands. As expected, the mean OD ratios for sestrin 2 in the LV-sestrin 2 group were significantly higher than those of the BC and LV-NC control groups (*p* < 0.05), while no significant difference in sestrin 2 protein expression was found between these last two groups (*p* > 0.05).

### Sestrin 2 overexpression decreases endogenous production of ROS *in vitro*

To examine the effect of sestrin 2 overexpression on ROS production, intracellular ROS levels were measured using 2′-7′-dichlorofluorescein diacetate (DCFH-DA) probe. As shown in Figure 1D, the relative fluorescence intensity in the LV-sestrin 2 group decreased significantly compared to the BC and LV-NC control groups (*p* < 0.05). There was no significant difference between the BC and LV-NC groups.

### Sestrin 2 overexpression decreases proliferation and increases apoptosis *in vitro*

**Figure 2:**
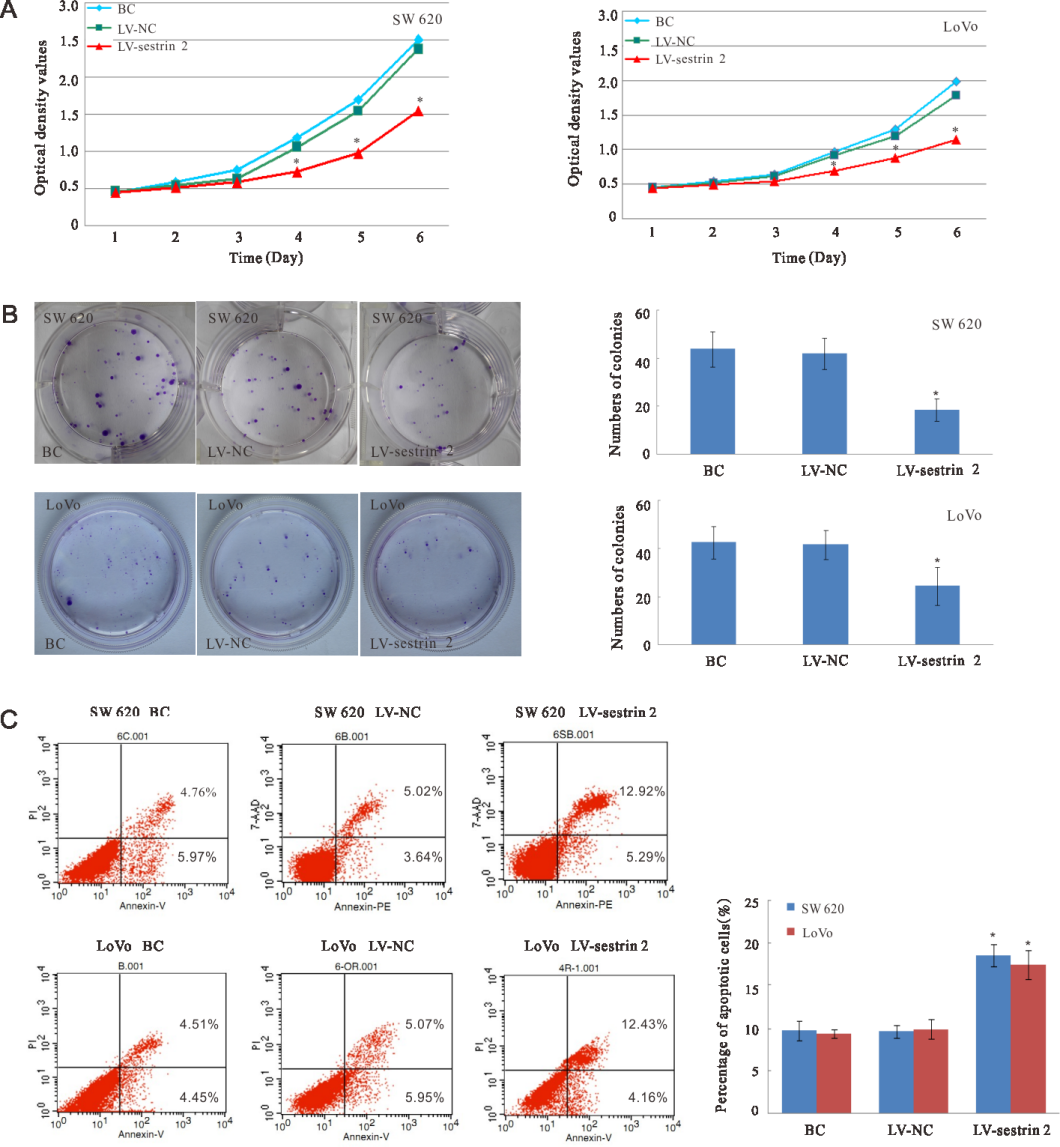
Effects of sestrin 2 overexpression on cell proliferation and apoptosis in CRC cells (**A**) Cell growth curves indicate significantly reduced growth in LV-sestrin 2 cells compared with cells in the BC and LV-NC control groups. (**B**) Plate clone formation assay. Colony numbers were significantly decreased in SW620 and LoVo cells in the LV- sestrin 2 group, compared with the BC and LV-NC groups. (**C**) Apoptosis assay. The percentage of apoptotic cells was significantly increased in the LV-sestrin 2 group compared with the BC and LV-NC groups. “BC”, blank control group; “LV-NC”, negative control group; “LV- sestrin 2”, sestrin 2 overexpression group. *p < 0.05 indicates statistically significant differences between LV-sestrin 2 group and BC or LV-NC control groups.

To determine the effects of sestrin 2 overexpression on cell proliferation, the CCK-8 cell proliferation assay was performed daily between days 1 and 6 post-transduction (Figure 2A). Repeated measures ANOVA revealed that proliferation was significantly reduced in both SW620 and LoVo cells overexpressing sestrin 2 at 4, 5, and 6 days (*p* < 0.05), compared with cells in the BC and LV-NC groups. There was a significant main effect of time (*p* < 0.05), but no significant time × group interaction was observed, indicating that the growth of both SW620 and LoVo cells transduced with sestrin 2 decreased progressively over time, but the main effect of time did not affect the differences observed between groups. In plate colony formation assays, the number of colonies of SW620 and LoVo cells was significantly lower (*p* < 0.05) in the LV- sestrin 2 group than in the BC and LV-NC groups (Figure 2B).

To determine whether sestrin 2 overexpression led to growth inhibition due to enhanced apoptosis, apoptosis ratios were analyzed by flow cytometry. The results showed that the percentage of apoptotic SW620 and LoVo cells was significantly increased (*p* < 0.05) in the LV-sestrin 2 group, compared with control BC and LV-NC groups (Figure 2C).

### Effects of sestrin 2 overexpression on proliferation, apoptosis, and sestrin 2 signaling protein profiles

**Figure 3:**
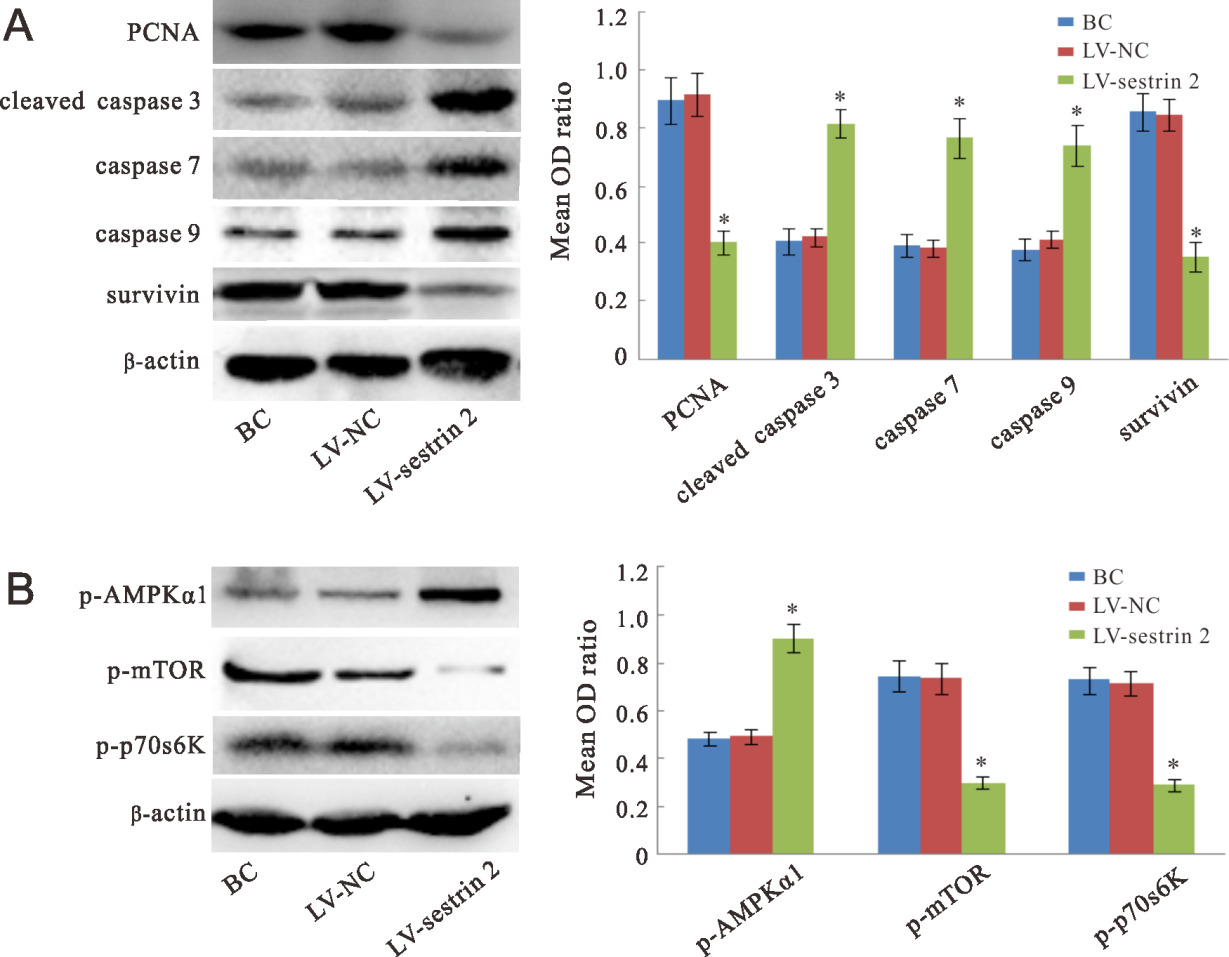
Western blot analyses Expression of cell proliferation- and apoptosis-related proteins (**A**), and proteins involved in the sestrin 2 signaling pathway (**B**) in SW620 cells. “BC”, blank control group; “LV-NC”, negative control group; “LV-sestrin 2”, sestrin 2 overexpression group. *p < 0.05, statistically significant differences between LV-sestrin 2 group and the control groups.

The expression of PCNA (a cell proliferation marker), caspase 3, caspase 7, caspase 9, and survivin (apoptosis markers), and p-AMPKα1, p-mTOR and p-p70s6K (proteins involved in the sestrin 2 signaling cascade) was studied by western blot in SW620 cells. Compared with the BC and LV-NC groups, the expression of PCNA and survivin proteins was significantly decreased (*p* < 0.05), while the expression of caspase 3, caspase 7, and caspase 9 was significantly increased (*p* < 0.05) in the LV-sestrin 2 group (Figure 3A). In addition, the expression of p-AMPKα1 was significantly increased (*p* < 0.05), whereas that of p-mTOR and p-p70s6K was significantly decreased (*p* < 0.05), in the LV-sestrin 2 group as compared with both control groups (Figure 3B).

### AMPK inhibition prevents changes in cell proliferation and apoptosis induced by sestrin 2 overexpression in CRC cells

**Figure 4:**
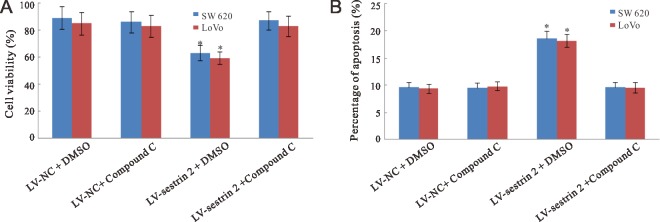
Effects of AMPK inhibition on cell proliferation and apoptosis in CRC cells (**A**) Compound C treatment increases cell viability in sestrin 2-overexpressing cells. (**B**) Compound C treatment decreases apoptosis in sestrin 2-overexpressing cells. *p < 0.05 indicates statistically significant differences among the groups.

To assess whether sestrin 2 overexpression-mediated AMPK activation influences proliferation and apoptosis, control and sestrin 2-transduced SW620 and LoVo CRC cells were treated with compound C (6-[4-(2-Piperidin-1-ylethoxy) phenyl]-3-pyridin-4-ylpyrazolo[1,5-a] pyrimidine), an inhibitor of AMPK. As shown in Figure 4A, compound C treatment rescued cell viability in sestrin 2-overexpressing cells, while no changes were observed after AMPK inhibition in control LV-NC cells with or without compound C treatment.

In terms of apoptosis, compound C exposure also normalized the apoptotic cell rate in sestrin 2-overexpressing CRC cells, while it had no effect on control LV-NC cells (Figure 4B).

### AMPK inhibition prevents changes in p-mTOR, p-p70s6K and survivin expression mediated by sestrin 2 overexpression in CRC cells

**Figure 5:**
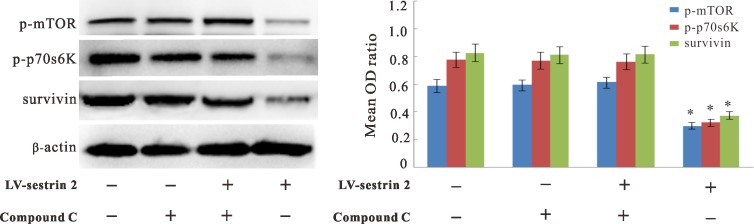
Effects of AMPK inhibition on p-mTOR, p-p70s6K, and survivin expression in CRC cells Inhibition of AMPK by treatment with compound C reversed protein expression/phosphorylation changes induced by sestrin 2 overexpression in CRC cells. *p < 0.05 indicates statistically significant differences among groups.

To assess whether AMPK activation also affects the decrease in p-mTOR, p-p70s6K, and survivin elicited by sestrin 2 overexpression, western blot analysis was conducted in SW620 CRC cells treated with compound C. Data showed that the levels of these three proteins were increased in the LV-sestrin 2 group after AMPK inhibition, while no changes were elicited by compound C treatment in control LV-NC cells (Figure 5).

### Sestrin 2 overexpression in CRC cells inhibit tumor growth *in vivo*

**Figure 6:**
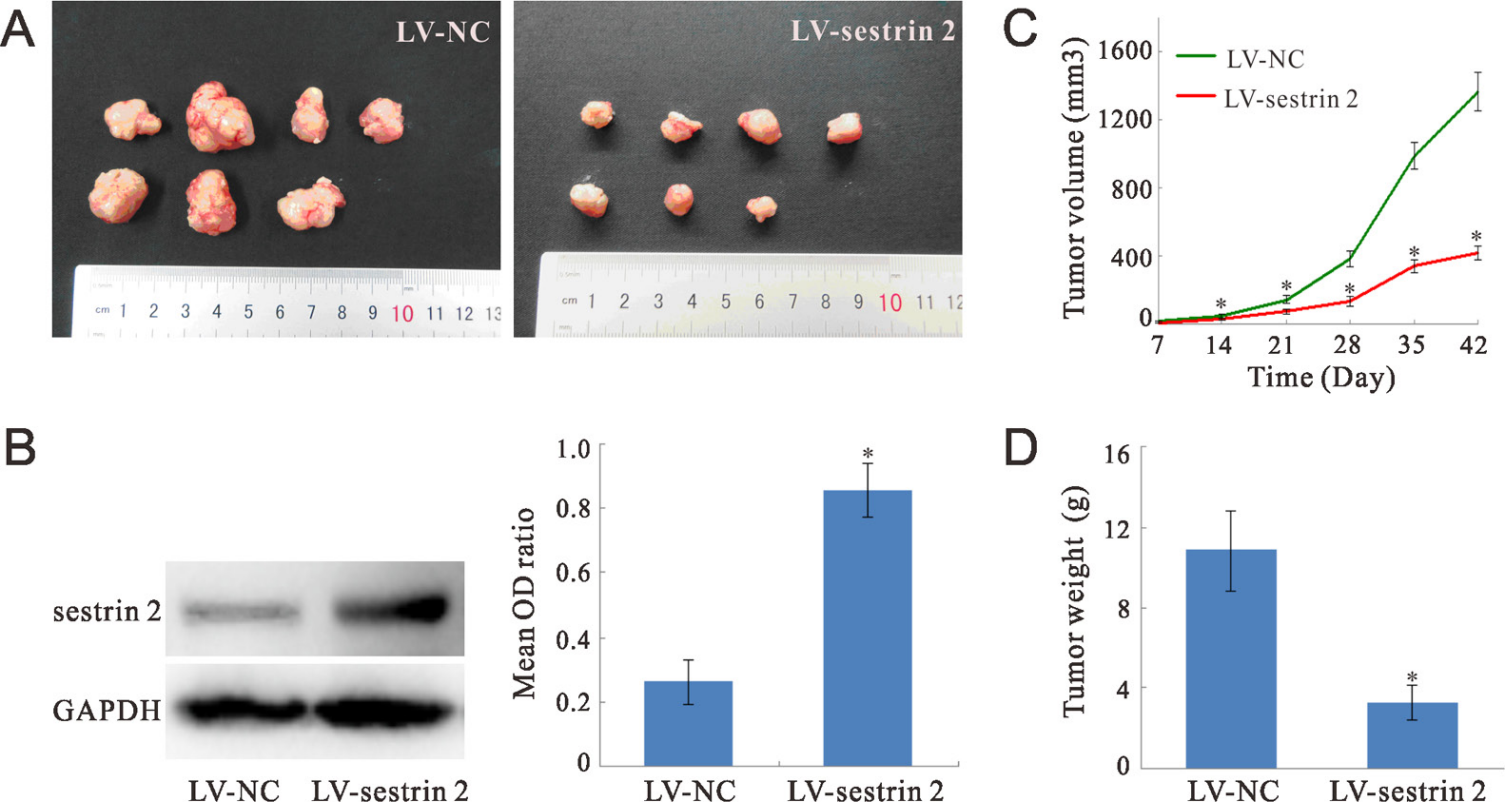
Sestrin 2 overexpression inhibits CRC xenograft growth in nude mice (**A**) Representative tumor xenografts from nude mice sacrificed 7 weeks after injection with CRC SW620 cells. (**B**) Representative western blots of sestrin 2 expression in excised tumors. The mean OD ratio represents sestrin 2 immunoreactivity relative to GAPDH immunoreactivity (*n* = 7 mice per group). (**C**) Tumor volume was monitored over time, 14 to 42 days after injection of SW620 CRC cells. (**D**) Weight of tumor xenografts excised 7 weeks after injection of SW620 cells. *p < 0.05 indicates statistically significant differences between the two groups.

To evaluate the tumor-suppressing ability of sestrin 2 *in vivo*, sestrin 2-overexpressing and control SW620 CRC cells were used to generate tumor xenografts in nude mice (Figure 6A).

Western blot analysis of xenograft extracts (*n* = 7 mice per group) obtained at sacrifice (7 weeks after tumor cell injection) showed that the expression of sestrin 2 was significantly increased (*p* < 0.05) in sestrin 2-transduced cells, compared to cells transduced with empty viral vectors (Figure 6B).

Tumor volume, monitored every week between days 14 and 42 after tumor cell implantation, decreased significantly over time in sestrin 2-overexpressing xenografts (Figure 6C). There was a significant main effect of time (*p* < 0.05), but no significant time × group interaction was observed, suggesting a true tumor volume reduction over time in sestrin 2 xenografts. Tumor weight, also recorded at sacrifice 7 weeks after SW620 cell injection, was significantly lower (*p* < 0.05) in sestrin 2-overexpressing xenografts compared with negative controls (Figure 6D).

## DISCUSSION

Cancer is considered a disease of deregulated cell proliferation and survival [37]. In this study we show that SW620 and LoVo CRC cells overexpressing sestrin 2 had lower proliferation ability and colony formation capacity than empty-vector transduced or non-transduced control cells. In this regard, we found that the expression of PCNA, a cell cycle regulatory protein, was downregulated upon overexpression of sestrin 2 in SW620 CRC cells [38]. Previous research has reported that sestrins inhibit cell proliferation in various human cancer cell lines such as H1299 lung carcinoma, MCF7 breast carcinoma, HT1080 fibrosarcoma, as well as in human immortalized fibroblasts [12]. Also, evidence showed that sestrin 2 could inhibit cell proliferation by arresting cells in the G1 phase of the cell cycle [39]. Notably, we observed that overexpressing sestrin 2 in CRC cells implanted in nude mice reduced both the weight and the size of the resulting tumors, indicating that sestrin 2 has tumor inhibiting properties *in vivo* as well as *in vitro*. Similarly, it was reported that sestrin 2-silenced lung carcinoma A549 tumor xenografts in athymic mice grow faster, as observed for p53-deficient cells [40].

Our previous study in CRC patients found that sestrin 2 was downregulated in neoplastic tissues, and correlated with advanced tumor stage as well as lymphatic and vascular invasion [16]. Thus, the role of sestrin 2 in CRC might be strengthened by its ability to restrict cell proliferation, therefore preventing tumor expansion and invasion.

Survival of tumor cells requires a continuous input of survival and trophic signals to suppress cell death or apoptosis. Thus, deregulated cell proliferation together with obligate compensatory suppression of apoptosis constitute the minimal common platform upon which all neoplasms progress [37]. The physiological function of sestrin 2 in cells is not restricted to the regulation of proliferation. For instance, sestrin 2 has been shown to modulate cell viability in response to stress, aggravating cell death in response to DNA-damage, but supporting cell viability in conditions of hypoxia [12]. The same study also reported that overexpression of sestrin 2 stimulated cell death in human embryonic kidney cells 293 [12]. Our results showed that overexpression of sestrin 2 in CRC cells up-regulated the expression of caspases 3, 7, and 9, which are principal mediators of apoptosis [41] and increased the percentage of apoptotic cells. Moreover, it also decreased the expression of survivin, a protein that inhibits caspases and blocks cell death. Survivin is highly expressed in most human cancers and is associated with poorer clinical outcomes [42–43]. Therefore, although the specific mechanism is not clear, we speculate that sestrin 2 may increase sensitivity to cell death in CRC cells by reducing the activation of the antiapoptotic protein survivin.

Potential tumor-suppressing effects of sestrin 2 are thought to arise from its ROS detoxifying actions and its ability to regulate AMPK/mTOR signaling [15, 17–20]. Hypoxic tumor microenvironments are associated to increased ROS levels, [35], a factor that may promote the development of CRC [36]. Here we found that sestrin 2 overexpression decreased the endogenous production of ROS *in vitro*, which suggests that inhibiting ROS overproduction by sestrin 2 overexpression may provide therapeutic benefits in CRC. Contributions of sestrin 2 to regulation of the AMPK/mTORC1 signaling pathway during tumor progression, by affecting protein synthesis, cell growth, and proliferation, have been widely documented [8, 24, 44]. However, whether sestrin 2 levels in CRC modulate AMPK/mTORC1 signaling is not known. In this study, we found that the expression of p-AMPKα1 was significantly higher in SW620 CRC cells overexpressing sestrin 2, while p-mTOR and p-p70s6K proteins were significantly decreased. p70s6K is a direct substrate of mTORC1, and its activation by phosphorylation can stimulate ribosome synthesis, accelerate mRNA translation, shorten the cell cycle, and promote cell growth [45]. Thus, we speculate that sestrin 2 may counteract CRC growth via activation of AMPK, thereby down-regulating the mTORC1 pathway. To address our hypothesis, compound C was used to inhibit the function of AMPK and block mTORC1 targets in SW620 CRC cells overexpressing sestrin 2. Interestingly, our results showed that the reduction in in cell viability, the increase in apoptosis, as well as the decrease in p-mTOR, p-p70s6K, and survivin expression elicited by sestrin 2 overexpression were attenuated or abrogated after AMPK inhibition with compound C. Thus, our findings indicate that sestrin 2 inhibits mTORC1 activity via AMPKα1, and down-regulates survivin expression in CRC cells.

In summary, our study demonstrated that sestrin 2 overexpression suppresses proliferation and activates apoptosis in CRC cell lines, and also inhibits the growth of CRC xenografts in nude mice. This suggests that sestrin 2 has anti-tumor effects on human CRC cells both *in vitro* and *in vivo*. Furthermore, our results strongly suggest that the reduction in sestrin 2 levels in human CRC samples, as reported by us [REF 16], may contribute to CRC pathogenesis via regulation of the AMPK/mTORC1 pathway. Specifically, our data suggests that sestrin 2 regulates proliferation and apoptosis through AMPK activation, and activated AMPK inhibits mTORC1 activation and phosphorylation, inhibiting in turn survivin function. We hope that this study will provide the basis for identification of molecular targets for therapeutic intervention in CRC. However, further research is required to clarify the detailed regulatory mechanisms responsible for the interaction of sestrin 2 with the AMPK/ mTORC1 signaling cascade in CRC development.

## MATERIALS AND METHODS

### Cell culture and antibodies

Human colon cancer SW620 and LoVo cell lines were purchased from the Shanghai Cell Bank at the Chinese Academy of Sciences (Shanghai, China). The cell lines were cultured in Leibovitz's L-15 medium (Gibco, Grand island, NY, USA) supplemented with 10% fetal bovine serum (FBS) (HyClone, Shanghai, China) and 2% penicillin/streptomycin (Beyotime, Jiangsu, China) at 37°C in a humidified atmosphere.

### Lentiviral vector-mediated overexpression of sestrin 2

EGFP-encoding lentiviral constructs for sestrin 2 overexpression were manufactured by Sunbio Medical Biotechnology Co., Ltd. (Shanghai City, P.R. China). The cDNA sequence for human sestrin 2 was as follows: forward 5′ -GAG GAT CCC CGG GTA CCG GTC GCC ACC ATG ATC GTG GCG GAC TCC-3′; reverse 3′-TCA CCA TGG TGG CGA CCG GGG TCA TGT AGC GGG TGA TGG-5′. SW620 and LoVo cells were seeded in 6-well plates at a concentration of 0.5 × 10^5^ cells per well (20–30% confluence) one day before transduction. Lentiviral vectors were transduced into cells at a multiplicity of infection (MOI) of 90 (SW620) or 20 (LoVo) using polybrene (10 μg/ml) and Enhanced Infection Solution (Genechem, China). At the same time, a non-targeted, control lentiviral vector expressing EGFP (Genechem, China) was transduced into cells using the same methods to control for the impact of the viral vector. After incubation for 12 h, the medium was replaced with fresh L-15 medium. SW620 and LoVo cells with stable sestrin 2 overexpression were designated as the LV-sestrin 2 group. SW620 and LoVo cells infected with non-target lentiviral vectors (negative controls) were designated as the LV-NC group. Untransfected SW620 and LoVo cells (blank controls) were designated as the BC group.

Transduction efficacy was initially assessed by fluorescence microscopy 3 days after transduction. At the indicated time points, cells were harvested for mRNA and protein analyses, as well as for other assays.

### Intracellular ROS generation assay

A reactive oxygen species assay kit (Beyotime, Jiangsu, China) was used to measure intracellular ROS according to the manufacturer's instructions. After incubation in 10 μM DCFH-DA-containing medium at 37°C for 20 min, cells were washed with PBS three times and resuspended in L-15 medium. The fluorescence intensity of the cell suspension was immediately measured by fluorescence-activated cell sorting (FACS) analysis. The values were expressed as a percentage of fluorescence intensity relative to blank control cells.

### AMPK inhibition assay

After 48 hours of successful transduction, SW620 and LoVo cells in both the LV-sestrin 2 and LV-NC and groups were treated with vehicle (DMSO; Sigma–Aldrich, USA) or 10 μM of compound C [46] (Sigma–Aldrich, USA) in DMSO for 48 hours. The SW620 and LoVo cells in the four groups thus established were then collected for cell viability, flow cytometry, and protein analyses.

### Cell proliferation assay

Cell viability was evaluated using Cell Counting Kit-8 (CCK-8; Beyotime, Jiangsu, China) according to the manufacturer's instructions. After lentiviral transduction (except for non-transduced cells), cells in the logarithmic growth phase were seeded in 96-well plates in triplicate at densities of 3 × 10^3^ cells per well. Cell proliferation was examined at 0, 1, 2, 3, 4, 5, and 6 days. In brief, 20 ul of CCK-8 (5 mg/ml in PBS) in 80 ul medium was added to each well, and the cells were incubated for 4 h at 37°C. Then, 160 μl of DMSO (Sigma–Aldrich, USA) were added to each well to stop the reaction. The plates were shaken for 10 min, and optical density values were measured in a spectrophotometer (Bio-Rad, Hercules, CA, USA) at 490 nm.

### Plate clone formation assay

Control and sestrin 2-transduced SW620 and LoVo cells were trypsinized, counted and seeded into 60 mm culture dishes at a density of 100 cells per dish in regular culture medium. After 15 days, cells were washed with PBS, fixed in 10% methanol for 15 min, and stained with Giemsa for 10 min. Visualized colonies were then photographed and scored. Each plate colony formation experiment was repeated at least three times.

### Flow cytometry analysis

An Annexin V-PE and 7-AAD (7-amino-actinomycin D) double-staining Apoptosis Detection kit (KeyGEN, Nanjing, China) was used to detect apoptotic activity according to the manufacturer's instructions. All experiments were performed 3 times. After the indicated treatments, the cells were subjected to FACS analysis within an hour using CellQuest software version 3.3.

### Real-time quantitative PCR analysis

Sestrin 2 mRNA expression levels in CRC SW620 and LoVo cells were analyzed by real-time quantitative PCR (qRT-PCR) analysis. β-actin expression was used as an internal control. The following primer sequences were used: for sestrin 2, forward 5′ -GAG GAT CCC CGG GTA CCG GTC GCC ACC ATG ATC GTG GCG GAC TCC-3′; reverse 3′-TCA CCA TGG TGG CGA CCG GGG TCA TGT AGC GGG TGA TG; for β-actin, forward 5′-ACG GTC AGG TCA TCA CTA TCG-3′, reverse5′- GGC ATA GAG GTC TTT ACG GATG-3′. Total cellular RNA was extracted using Trizol reagent (Takara Bio, Dalian, China) following the manufacturer's protocol. RNA concentration and purity were assessed using a UV spectrophotometer (Ultrospec 2100 Pro, Amersham, USA). Total RNA was reverse-transcribed using the PrimeScript RT Reagent kit (TAKARA Dalian, China). qRT-PCR reactions were performed in a 96-well plate using a 35-cycle, 2-step PCR protocol in an iQ Multiplex Powermix system (Bio-Rad, USA). Amplification steps were performed using CFX Manager software version 1.6 (Bio-Rad, USA). A melting curve was generated, and amplification curves were analyzed at the end of the amplification process. The threshold cycle (Ct) was used to determine the relative expression of each gene, and relative mRNA expression levels were calculated using the 2^−(△C sample-△C control)^ method.

### Western blot analysis

After the indicated treatments, total protein was extracted from cells using the Total Protein extraction kit (Keygen Biotech, Nanjing, China) according to the manufacturer's instructions. In brief, the cells were harvested and then lysed in lysis buffer containing 150 mM sodium chloride, 0.1 M Tris, 1% Tween-20, 50 mM diethyldithiocarbamic acid, 1 mM ethylenediamine tetraacetic acid, and protease inhibitors at pH 8.0. The lysates were centrifuged at 12,500 rpm at 4°C for 15 min, and the supernatants were collected. Protein concentrations were measured using a bicinchoninic acid (BCA) protein assay (Pierce, USA) following the manufacturer's instructions. Electrophoresis was performed using a Mini-Protean system (Bio-Rad Laboratories, USA). The protein extracts were separated by sodium dodecyl sulfate-polyacrylamide gel electrophoresis (SDS-PAGE) and electrotransferred onto an Immobilon polyvinylidene difluoride (PVDF) membrane (Millipore Corporation, USA) using an electrophoretic transfer system (Bio-Rad Laboratories, USA). The PVDF membranes were blocked in freshly prepared buffer supplemented with 5% nonfat dry milk (Boster Bioengineering, Wuhan, China) for 1 h at 37°C. A mouse anti-sestrin 2 monoclonal antibody (1:200, Santa Cruz, USA), a rabbit anti-proliferating cell nuclear antigen (PCNA) monoclonal antibody (1:1000, Epitomics, USA), a rabbit anti-survivin monoclonal antibody (1:2000, Epitomics, USA), a rabbit anti-caspase 9 monoclonal antibody (1:1000, Epitomics, USA), a rabbit anti-caspase 7 monoclonal antibody (1:1000, Epitomics, USA), a rabbit anti-cleaved-caspase 3 monoclonal antibody (1:1000, Abcam, USA), a rabbit anti-p-AMPKα1 monoclonal antibody (1:1000, Abcam, USA), a rabbit anti-p-mTOR monoclonal antibody (1:1000, Abcam, USA), a rabbit anti-p-p70s6K polyclonal antibody (1:1000, Abcam, USA), a mouse anti-GAPDH monoclonal antibody (1:2000, Epitomics, USA), and a rabbit anti-β-actin monoclonal antibody (1:1000, Sigma–Aldrich, USA), were used as primary antibodies for western blot studies. The PVDF membranes were then incubated with the primary antibodies overnight at 4°C, washed with a mixture of Tris buffered saline and Tween-20 (TBST), and incubated with a horseradish peroxidase (HRP)-conjugated secondary antibody in TBST (1:5,000 dilution, goat anti-rabbit IgG-HRP or goat anti-mouse IgG-HRP; Santa Cruz) for 60 min at 37°C. Protein bands were visualized using an enhanced chemiluminescence substrate kit (Pierce, USA) before digital scanning (Bio-Rad Laboratories). Image pixel density was quantified using Quantity One software (Bio-Rad Laboratories).

### Animal studies

Five-week-old female BALB/c nude mice were obtained from the Laboratory Animal Center of Chongqing Medical University. These mice were maintained in a specific pathogen-free unit under isothermal conditions. All experimental procedures were conducted in accordance with the Guide for the Care and Use of Laboratory Animals (National Institutes of Health). SW620 and LoVo cells (3 × 10^6^) transduced with sestrin 2-encoding lentivirus or a negative control vector were suspended in 100 μl of PBS. The cells were then implanted subcutaneously into the right flank of nude mice to form tumor xenografts. When tumor size reached approximately 100 mm^3^ (after ∼2 weeks), tumor diameters were measured every week using Vernier calipers. Tumor volume was calculated using the following formula: volume (mm^3^) = length × width^2^/2. After 8 weeks, the tumors were harvested for further analyses.

### Data analysis

Data are expressed as means ± standard deviation (SD). Student's *t*-test was used for statistical analyses of differences between two groups (SPSS 17.0). Differences between groups were also determined using one-way analysis of variance (ANOVA) followed by Tukey's honest significant difference (HSD) post hoc multiple comparison test (SPSS 17.0). Repeated measures ANOVA was used to compare group means of a dependent variable across repeated measurements of time (SPSS 17.0). A value of *p* < 0.05 was considered statistically significant. All experiments were repeated in triplicate.
